# Soil organic matter and the extracellular microbial matrix show contrasting responses to C and N availability

**DOI:** 10.1016/j.soilbio.2015.05.025

**Published:** 2015-09

**Authors:** M.A. Redmile-Gordon, R.P. Evershed, P.R. Hirsch, R.P. White, K.W.T. Goulding

**Affiliations:** a Rothamsted Research, Harpenden, Herts AL5 2JQ, UK; b Organic Geochemistry Unit, Bristol Biogeochemistry Research Centre, School of Chemistry, University of Bristol, BS8 1TS, UK

**Keywords:** EPS protein dynamics, Exocellular peptide, Biophysical interface, Labile substrate C:N ratio, Exopolysaccharide, Biofilm amino acids

## Abstract

An emerging paradigm in soil science suggests microbes can perform ‘N mining’ from recalcitrant soil organic matter (SOM) in conditions of low N availability. However, this requires the production of extracellular structures rich in N (including enzymes and structural components) and thus defies stoichiometric expectation. We set out to extract newly synthesised peptides from the extracellular matrix in soil and compare the amino acid (AA) profiles, N incorporation and AA dynamics in response to labile inputs of contrasting C/N ratio. Glycerol was added both with and without an inorganic source of N (10% ^15^N labelled NH_4_NO_3_) to a soil already containing a large pool of refractory SOM and incubated for 10 days. The resulting total soil peptide (TSP) and extracellular pools were compared using colorimetric methods, gas chromatography, and isotope ratio mass spectrometry. N isotope compositions showed that the extracellular polymeric substance (EPS) contained a greater proportion of products formed *de novo* than did TSP, with hydrophobic EPS-AAs (leucine, isoleucine, phenylalanine, hydroxyproline and tyrosine) deriving substantially more N from the inorganic source provided. Quantitative comparison between extracts showed that the EPS contained greater relative proportions of alanine, glycine, proline, phenylalanine and tyrosine. The greatest increases in EPS-peptide and EPS-polysaccharide concentrations occurred at the highest C/N ratios. All EPS-AAs responded similarly to treatment whereas the responses of TSP were more complex. The results suggest that extracellular investment of N (as EPS peptides) is a microbial survival mechanism in conditions of low N/high C which, from an evolutionary perspective, must ultimately lead to the tendency for increased N returns to the microbial biomass. A conceptual model is proposed that describes the dynamics of the extracellular matrix in response to the C/N ratio of labile inputs.

## Highlights

• Extracellular polymeric substances (EPS) increased with labile C availability.
• EPS production efficiency decreased with C/N ratio of available substrate.
• A conceptual model for the EPS response to C/N ratio of inputs is proposed.
• High e-polysaccharide was coupled with improved extracellular peptide extraction.
• Colorimetric analysis of protein reflected similar trends given by GC/FID.

## 1 Introduction

It has been estimated that at some stage in the life-cycle over 90% of microbial species will exist in a biofilm (Vu et al., 2009). While the characteristically macroscopic biofilms such as those found in nutrient rich watercourses or chemostats (Flemming and Wingender, 2010) are unlikely to be ubiquitous in oligotrophic soil, biological aggregates have been observed in soils given an exogenous carbon source (Nunan et al., 2003). The extracellular matrix is suspected to provide various benefits, such as improving the availability of water between hydration cycles (Or et al., 2007a), maintaining colony adhesion to soil aggregates (Or et al., 2007b) improving soil aggregate stability (Redmile-Gordon et al., 2013) and improving the efficiency of enzyme activity and nutrient capture (Flemming and Wingender, 2010). Understanding biofilm dynamics in response to nutrient availability is therefore not only of fundamental interest, but of functional importance in agricultural soils, particularly for rhizosphere biology and soils receiving organic inputs. Characterising biofilm dynamics requires an understanding of the production of extracellular polymeric substance (EPS) which connects individual cells to surfaces or to one-another in a biofilm. Contrary to common belief, EPS is composed of more than just polysaccharides, including a wide variety of proteins and glycoproteins (Flemming et al., 2007), and considerable quantities of extracellular DNA (Pietramellara et al., 2009; Dominiak et al., 2011). There is an increasing realisation that the factors influencing the dynamics of EPS are crucial for the development of more accurate environmental models describing nutrient transfer (Flemming et al., 2007).

Films of EPS can support a high level of enzyme activity (Flemming et al., 2007). Both leu-aminopeptidase and β-glucosaminidase were found to be retained in the EPS of aquatic biofilms (Romani et al., 2008). This retention is advantageous to prevent enzymes and products of catalysis from being lost to solution and environment, thus improving the efficiency of enzyme activity (Flemming and Wingender, 2010). We postulate that factors governing the production of EPS-peptides in soil will be of further relevance to the turnover of soil organic matter (SOM). Burns et al. (2013) presented a firm case that soil peptide research should embrace biofilm dynamics to complement future directions in soil enzyme research. This case was visionary because at that time, the selective extraction of peptides from the soil extracellular matrix had not yet been demonstrated.

Until now, the EPS extracted from soil has been characterised only broadly, for example as ‘EPS-protein’ or ‘EPS-polysaccharide’. The proteinaceous component of EPS is multifunctional, fulfilling structural, adhesive and enzymic roles (Flemming and Wingender, 2010). A method to determine the EPS exuded from the soil microbial biomass was recently proposed by Redmile-Gordon et al. (2014). In agreement with Nunan et al. (2003), who used microscopy to observe cell clusters, Redmile-Gordon et al. (2014) found that EPS-polysaccharide in an oligotrophic soil was increased through the provision of labile C (glycerol). The C was provided without any N, and in conjunction with drought stress which is expected to cause increased exudation of EPS (e.g. Roberson and Firestone, 1992; Kakumanu et al., 2013). However, Redmile-Gordon et al. (2014) did not find any statistically significant increases in EPS-protein after extraction and proposed this was due to the unusually low N availability in the fallow soil: managed as zero-input for >30 years (inorganic N < 0.1 μg g^−1^; organic N < 0.03%). Two questions remained unanswered: a) was extraction with cation exchange resin sensitive enough to detect increases in exudation of EPS-protein in soil? and, b) how did the nutrient quality (C/N ratio of labile substrate and soil organic matter content) affect the exudation of proteinaceous EPS?

The C/N ratio of inputs to soil has also been the subject of much debate in respect of the effect on SOM turnover, the microbial biomass and nutrient dynamics (e.g. Blagodatskaya and Kuzyakov, 2008). Simple stoichiometry predicts that N must be freely available to support the anabolic process of protein translation. The bulk of organic N contained within soil humus was found to be in amide/peptide structures (Fruend et al., 1994) which suggests it could potentially be made available via enzymic activity. Numerous authors have since postulated that soil microbes can perform ‘N-mining’ of recalcitrant soil organic matter (SOM), acquiring N through a cascade of exoenzyme activities when given substrate(s) with a high C/N ratio (e.g. Blagodatskaya and Kuzyakov, 2008; Fontaine et al., 2011). Factors affecting the mobility and activity of enzymes such as the hydration status of the biophysical interface are expected to be important in SOM turnover (Burns et al., 2013). However, given the lack of research into the effect of C/N ratio of labile inputs on EPS production in soil, it is hard to predict how EPS and other SOM interact.

Drawing upon knowledge from related disciplines, high C availability has been linked to high production rates of EPS-polysaccharide over microbial biomass in monocultures of *Rhizobium tropici* (Staudt et al., 2012). Likewise, a high C/N ratio favoured EPS-polysaccharide over EPS-protein in a chemostat (Wang et al., 2014) and low C/N ratio caused disruption of the biofilm structure (Luo et al., 2014). However, the converse has also been found, with increased production of EPS-peptide occurring with low N availability. For example, Chen et al. (2012) found the highest EPS-protein content at a substrate C/N of 156. While the examples introduced here may provide clues for the dynamics of EPS in soil, no studies have yet compared the response of EPS and total soil peptides (TSP) to inputs of contrasting C/N availability.

A well-defined example of non-enzymic extracellular peptide structures which serve to improve the efficiency of suites of physically supported enzymes was discussed by Fontes and Gilbert (2010). Appropriately these structures are termed ‘scaffoldins’ and form part of the larger ‘cellulosome’ which is an extracellular multienzyme complex retained in close proximity to the cell, and contributes to total EPS. Protein to protein interactions regulate the structure in this example matrix to maximise the efficiency of cellulose catalysis. We propose that in soil, where low N causes an increase in production of EPS-peptides, these comprise contributions from a combination of exoenzymes and structural EPS components for improving enzyme efficiency. As a first step, the quantification of matrix amino acids provides information on overall peptide dynamics: encompassing enzymes, structural peptides and glycoproteins that respond to substrate regimes of contrasting stoichiometry. Given the multifunctional roles of EPS-peptides in the catalysis of organic matter (e.g. Fontes and Gilbert, 2010; You et al., 2012), the effect of the C/N ratio of inputs is likely to be of central importance not only for the emerging integrated focus on the microbial extracellular matrix (Redmile-Gordon et al., 2014), but also in the debate surrounding organic matter turnover (e.g. Kemmitt et al., 2008).

The use of ^15^N to trace substrate utilisation and peptide production *de novo* has previously helped enable comparative estimates of AA pools responding specifically to inputs. ^15^N/^14^N ratios (δ^15^N) can be used to determine the origin of N, i.e. products of native soil N vs. products of exogenous N (Knowles et al., 2010). We therefore decided to apply this technique to help reveal contrasting dynamics between EPS peptides vs. an estimate of those in the total SOM. The present study was designed to 1) test the EPS extraction technique's specificity for peptides produced *de novo*, 2) elucidate the responses of EPS-polysaccharide and EPS-peptide pools to contrasting input C/N ratios, 3) to compare patterns of ^15^N incorporation into EPS amino acids with those of the total soil peptide fraction, and 4) to propose a conceptual model for input dependent EPS-peptide dynamics in soil. The motivation for this model was to facilitate the incorporation of the EPS/biofilm concept into future studies of SOM.

## 2 Materials and methods

### 2.1 Soil treatments

Soil was sampled on 22nd February 2012 from the Highfield Reversion Experiment at Rothamsted Research. This soil is a Batcombe series (Soil Survey of England and Wales) fine silty loam, over clay drift with silicious stones; it is approximately equivalent to a Chromic Luvisol as defined in the FAO soil classification system. The pH of soil in water was 6.50, and sand, silt and clay proportions were 11, 66, and 23%, respectively (Watts and Dexter, 1997). Plot 12 (historically managed as grassland for 60 years and switched to arable management in 2008) was sampled randomly with a 2.5 cm auger to a depth of 23 cm and sieved (moist) < 2 mm. Visible root material and organisms were removed. This specific soil management reversion of grassland to arable was selected due to the intrinsic presence of a large pool of humified and refractory organic matter. The soil was pre-incubated at 25 °C in the dark for one week to allow the mineralisation of substrates brought into fresh contact with microorganisms during sieving. Total organic C and N content was 2.18% and 0.204%, respectively (C/N = 10.72).

Portions of moist soil (33 g oven dry weight at 39% of water holding capacity) were placed into 125 mL glass beakers to form 12 microcosms. C and N were provided as simple chemically defined substrates, respectively glycerol (C_3_H_8_O_3_) and ammonium nitrate (NH_4_NO_3_). Aqueous solutions of these were dripped onto the surface of each of 3 replicates. Treatment structure is provided in Table 1 and shows the extreme and contrasting bioavailable C/N environments created, with a control (‘0’) and labile substrate C/N ratios of < 1 (‘N’), 20 (‘CN’), and >100 (‘C’). All microcosms provided with N received 150 μg N g^−1^ soil as 10.1% ^15^N enriched NH_4_NO_3_ (equal label ammonium ^15^N/nitrate ^15^N; Europa Scientific, Electra Way, Crewe, Cheshire lot # RX2602). Soil moisture was brought to 60% of its water holding capacity (WHC) by addition of each amendment.

Microcosms were then placed in desiccators. Each desiccator formed 1 statistical block thus contained one microcosm of each treatment, and experimental units were randomly distributed within. A vial of soda lime was placed in the centre of each desiccator to prevent CO_2_ accumulation. Silica gel in the base of each desiccator (380 g dry weight) was changed every 48 h in order to progressively dry the soil, which favours the production of EPS (Roberson and Firestone, 1992). Soil was allowed to dry to 20% WHC before being re-moistened to 40% WHC with deionised water: this was required twice over the period of 10 days. Besides previously being observed to trigger an EPS response, moisture fluctuations enabled more complete circulation of solutes as would occur in a soil system exposed to rainfall etc.

### 2.2 EPS extraction and colorimetric analysis

After the removal of soluble microbial products from the soil with dilute CaCl_2_ (0.01 M), EPS was extracted using cation exchange resin as described by Redmile-Gordon et al. (2014). Three technical replicates or ‘subsamples’ (2.5 g dry weight equivalent) of each biological replicate were used to estimate microbial ATP before extraction and three were subjected to extraction of EPS. ATP concentrations of the extracted soils were compared with those in the non-extracted soils to enable an estimate of the extent of cell-lysis caused. ATP of the microbial biomass was quantified using the method described by Redmile-Gordon et al. (2011). EPS-polysaccharide content was estimated using the method of DuBois et al. (1956) and EPS-protein using a Lowry assay modified for use with soil extracts that contain a potentially confounding polyphenolic fraction (Redmile-Gordon et al., 2013).

### 2.3 Peptide hydrolysis and purification

From each technical replicate of EPS extract, 20 mL aliquots were taken, combined and freeze-dried to provide a lyophilate of 60 mL extract per biological replicate (or microcosm). Lyophilate was transferred to 10 mL thick-walled Pyrex digestion tubes (threaded apertures with PTFE sealed caps) using 3 successive 1 mL aliquots of 0.1 M HCl. The HCl was then removed under a stream of N_2_ at 60 °C. Norleucine (20 μg) was added to each EPS extract (and milled soil) to serve as an internal standard (Jim et al., 2003). AAs in the soil (TSP) and EPS were extracted by hydrolysis with 5 mL 6 M HCl at 100 °C for 18 h. Asparagine (Asn) and glutamine (Gln) could not be distinguished from their corresponding acids (Asp and Glu) as the amides were destroyed during hydrolysis (Roberts and Jones, 2008). The sums of original amides and deamidated AAs are thus referred to as Glx, and Asx, respectively. To prevent phthalate contamination, glassware was used exclusively, having been washed with detergent (Decon 90; Decon Laboratories Ltd.), rinsing with double distilled water (DDW) then baked in a furnace at 450 °C for ≥ 8 h.

Impurities were removed from the hydrolysate by passing it through H-saturated cation exchange resin (Dowex 50X-W8; Sigma Aldrich, Dorset, UK). Resin was pre-soaked for 12 h in 3 M NaOH to remove contaminants. NaOH was removed and the resin washed in DDW six times. To ensure all cation exchange sites were saturated with H^+^, the resin was steeped in 6 M HCl for ≥ 24 h. 1 mL aliquots of resin were transferred to flash columns, and washed (6 × 2 mL DDW). Each soil hydrolysate was added to a dedicated column for the exchange of AAs. Non-affixed solutes were eluted to waste with 4 × 2 mL DDW before displacing the AAs with 6 × 1 mL 2 M NH_4_OH. The resulting solution was blown down to dryness under a stream of N_2_ gas in a heating block set to 60 °C.

### 2.4 Derivatisation

A solution of AA standard was prepared containing 1 mg of each AA mL^−1^ 0.1 M HCl. The δ^15^N value of each AA had previously been determined by elemental analyser–isotope ratio mass spectrometer (EA–IRMS); all ratios were at approximately natural abundance. The standard solution was used to confirm peak identities via retention time and mass spectral comparisons, and as secondary standards for isotope ratio mass spectrometry. N-acetyl, O-isopropyl derivatives of hydrolysed AAs first involved conversion to their respective isopropyl esters followed by N-acetylation as described by Corr et al. (2007). Derivatives were purified by phase extraction with saturated aqueous NaCl and vortexing, the bulk of the organic phase was removed, combined, and all solvent was evaporated under a gentle stream of N_2_ at 20 °C. Any residual water was removed by addition of dichloromethane (DCM) and evaporation under N_2_ at 20 °C. Derivatised samples were capped, sealed with PTFE tape and stored in a freezer at −20 °C until required for analysis by gas chromatography with flame ionization detection (GC-FID) and gas chromatograph–combustion–isotope ratio mass spectrometry (GC–C–IRMS).

### 2.5 GC-FID analyses

Samples were dissolved in approximately 100 μl ethyl acetate to obtain an appropriate concentration of volatiles for GC peak symmetry and analysed using a Hewlett–Packard 5890 fitted with FID and VF-23MS capillary column (3-cyanopropylpolysiloxane stationary phase, 60 m × 0.32 mm internal diameter; 0.15 μm film thickness; Varian Ltd., Oxford, UK). 0.5 μL of each sample was introduced using direct on-column injection. The carrier gas was H_2_ with a flow rate of 3 mL min^−1^; head pressure 10 PSI. Initial oven temperature was 40 °C and held for 1 min before heating at 15 °C min^−1^ to 120 °C (no hold), then 3  °C min^−1^ to 190 °C (no hold) and finally 5 °C min^−1^ to 250 °C and held for 20 min. The concentration of each AA was calculated by comparison of peak area with the internal standard (norleucine) and adjusted for flame response of each AA calculated using the mixed standard.

### 2.6 GC–C–IRMS analyses

N stable isotope compositions of AAs were determined using a ThermoQuest Trace GC connected to a DeltaPlus XP via a GC combustion III interface. A similar VF-23MS capillary column was used as for GC-FID (see above). Samples were introduced to a PTV ‘splitless’ injector via auto-sampler. δ^15^N values needed no adjustment for addition of the derivative (NAIP) as it contains no nitrogen (Corr et al., 2007).

Analyte identities were verified using a GC–MS (ThermoFinnigan, trace-gc: Hemel Hempstead, UK) also equipped with a VF23-MS column, Helium carrier gas (2 mL min^−1^). Samples were introduced using a split/splitless injector in splitless mode, (inlet temp 250 °C). Source temperature was held at 200 °C, mass analyser scanning the range *m/z* 50–650. The GC oven was programmed as for GC-FID (described above). Mass spectra were consistent with those normally observed for NAIP-derivatives of the AA described (or isomers), i.e. the mass of the ionised amino acid NAIP minus mass-combinations of fragments: propyl [CH(CH_3_)_2_]/O-propyl [OCH(CH_3_)_2_]/COO-propyl [CO_2_CH(CH_3_)_2_], and/or one of acetyl [COCH_3_]/[CH_3_], and one or two H^+^. Elution order was confirmed using the standard mixture and followed that observed by Corr et al. (2007) using the same column type (VF-23MS).

### 2.7 Statistical analyses

The 4 treatments were analysed in a 2 × 2 factorial arrangement with 3 replicates. For direct comparison between the EPS peptide and total soil peptide (TSP) extracts we applied ANOVA (Genstat, v. 17, VSN international) to probe the experimental structure (blocks/treatment/extraction-method/amino-acid) and compare AA profiles between the two extracted fractions. For comparison of treatment effect upon the concentration of AA quantified within each type of extract (EPS or TSP), a separate ANOVA was applied to each extract as the previous pooled analysis above had confirmed EPS and TSP were indeed statistically different fractions. Since residual variability increased with AA concentration data, they were transformed (log_10_) to identify main effects. ANOVA was also used to compare AA concentrations in the EPS per unit microbial biomass (μg EPS-AA nmol^−1^ ATP). The residual errors of these data did not show the same systematic drift with concentration and thus did not require transformation.

## 3 Results

### 3.1 ^15^N incorporation into biogenic amino-acids of EPS vs TSP pool (GC-IRMS)

**Fig. 1:**
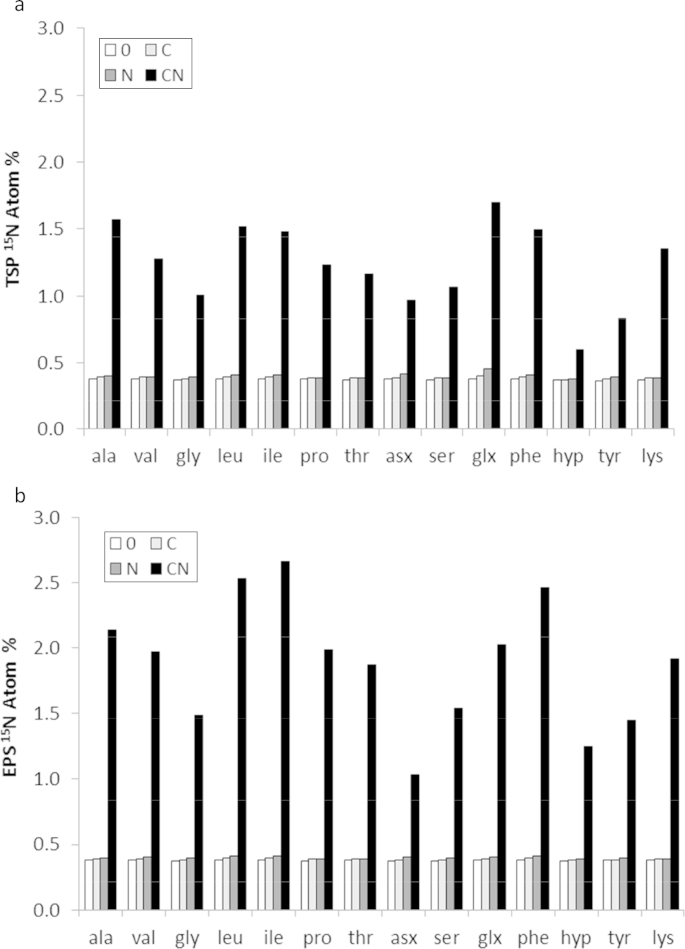
a: ^15^N incorporation into the total hydrolysable soil peptide fraction (TSP; l.s.d. = 0.041 at.%). b: ^15^N incorporation into the extracellular matrix (EPS; l.s.d. = 0.020 at.%).

After incubation, only the ‘CN’ treatment caused any substantial increase in ^15^N assimilation (Fig. 1a and b). Extraction method (EPS vs. TSP) had a highly significant effect on the isotope signature of AAs extracted (F_13,208_ = 376; p < 0.001) with EPS AAs being more enriched in ^15^N than those in the total soil (TSP), 0.76% compared to 0.60%, respectively (l.s.d. = 0.02 Atom%). This shows that the two fractions contain N with different origins and that TSP contains proportionally more N derived from the native pool of SOM.

### 3.2 ^15^N treatment effects (GC–IRMS)

For the total soil peptide fraction, a positive interaction was observed between C and N (F_1,6_ = 908; p < 0.001). The ‘CN’ treatment caused large and statistically significant increases of ^15^N in all amino acids (Fig. 1a) with the Glx pool (collectively Glu and Gln) being the most enriched. Much smaller enrichments occurred where ^15^N was given without C (from 0.38 % to 0.45% ^15^N), indicating the microbial biomass was C limited. Glx enrichment was still statistically significant (l.s.d. 0.041% ^15^N), but was the only AA group to incorporate statistically significant quantities of ^15^N when not given additional C.

In contrast to TSP, adding N alone caused no statistically significant enrichment of any AA in the EPS (Fig. 1b). A strong positive interaction was observed between C and N (F_1,6_ = 4217; p < 0.001) with treatment ‘CN’ causing statistically significant increases of ^15^N in all AA residues (Fig. 1b). Leucine (Leu) isoleucine (Ile) and phenylalanine (Phe) were the most ^15^N enriched AAs in the EPS. However, the greatest proportional increase in ^15^N enrichment in EPS (by comparison to TSP) was observed for hydroxyproline (Hyp), where the difference between treatment ‘0’ and ‘CN’ was 4 times greater than was seen in TSP (contrast Fig. 1a with 1b). Asx (collectively asparagine and aspartate) was the least enriched group found in the EPS extracts.

### 3.3 Quantitative AA determinations (GC–FID)

**Fig. 2:**
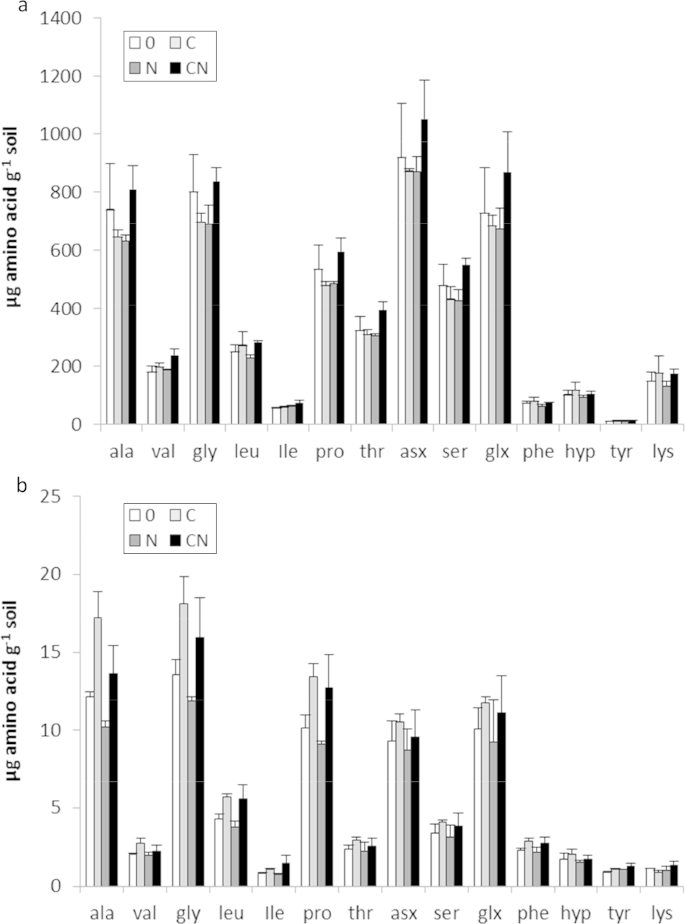
a: Quantification of TSP amino acids using GC-FID (±std. error). b: Quantification of EPS amino acids using GC-FID (±std. error).

From the 7 most abundant AA's measured in TSP (Fig. 2a) it might appear that i) unbalanced substrate provision (‘C’ or ‘N’ alone) caused a reduction in TSP, and ii) when provided together (as ‘CN’) that an increase in net AA production occurred. However, remaining true to the original experimental design (analysing treatment effects on all AAs combined), ANOVA of the logged concentrations showed that no consistent treatment effects occurred on the AA concentration in TSP (Table 2). In light of the contrast observed above, it became apparent that a clear divide had occurred at the median concentration (about 300 μg AA g^−1^ soil). The more abundant AAs showed an unbroken and consistent pattern of decline with unbalanced substrate addition (C without N, or N without C), and conversely an increase when C and N were provided together. In contrast, the 7 least abundant AAs showed response patterns more typical of the EPS (increasing only with C addition; Fig. 2b) which may indicate a larger contribution of EPS to the total soil peptide pool than the EPS extraction suggested. A *post-hoc* analysis of this contrast showed that the pattern of response was indeed different for the high and low subsets (F_1,104_ = 4.2; p = 0.043).

Unlike TSP, EPS-AA showed a strong and consistent link to treatment, specific to the addition of carbon (F_1,6_ = 9.32; p = 0.022). On average, C addition increased EPS AA concentrations by about 25% (Table 2). Though it may appear that a low C/N ratio (treatment N) caused decreases in the quantity of EPS-AA, no statistical significance was attributed by ANOVA to the depletive effect of N using this measure of concentration in *soil* (P = 0.35). However, the effect upon EPS-AA concentration per unit microbial biomass (presented in Section 3.5) was large and statistically significant. Of the 14 AAs quantified here, the extracted EPS typically accounted for less than 2% of TSP. There was no interaction between C and N when measured as a concentration in soil (p = 0.774). No significant third level effects were identified, i.e. the increases per AA were all proportionally similar in response to the glycerol-C provided.

### 3.4 Colorimetric analyses

**Fig. 3:**
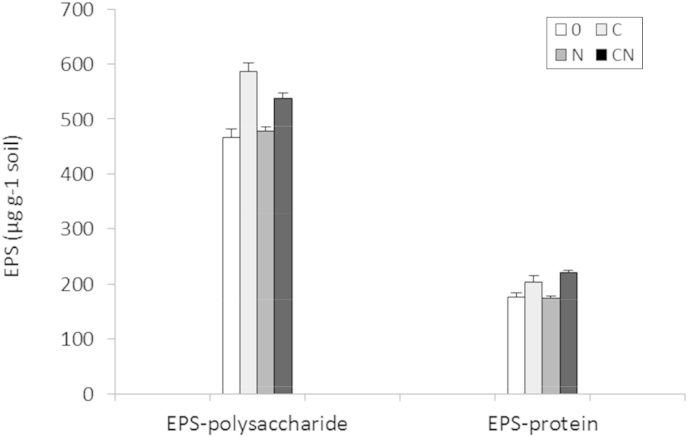
Total extracted EPS-protein and polysaccharide determined colorimetrically (±std. error).

ANOVA for C and N effects on EPS-protein data (Fig. 3) showed a consistently positive effect of glycerol-C (p < 0.001). As with GC-FID results (Section 3.3) N showed no statistically significant effect upon the total ‘EPS-protein’ as a concentration in soil (Fig. 3; P = 0.30). The response pattern of EPS-polysaccharide to treatment observed using colorimetric methods generally reflected that of individual AA responses (Fig. 2b). The main factor affecting the quantity of extracellular polysaccharide was C availability (p < 0.001). Importantly, a statistically significant interaction between C and N also occurred, lowering the concentration of EPS-polysaccharide when N was included (p = 0.033). Accordingly, treatment ‘C’ (highest C/N ratio) caused the greatest production of EPS-polysaccharide (Fig. 3; l.s.d. = 27.08 μg g^−1^ soil).

### 3.5 ATP analysis of the microbial biomass and EPS production efficiency

**Fig. 4:**
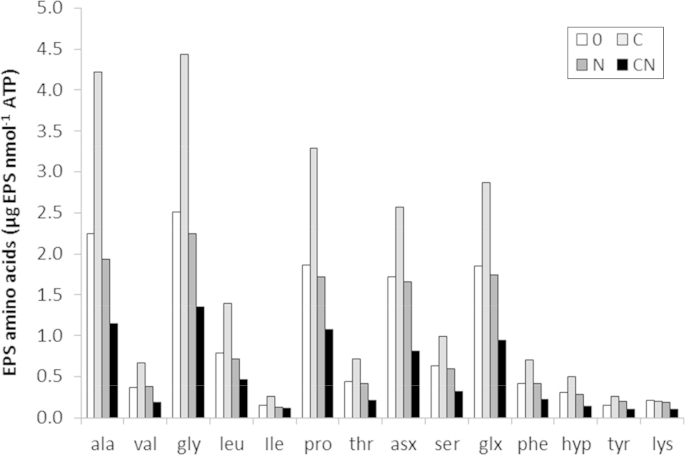
EPS-amino acid production efficiency. ANOVA of the additive effects of C (p = 0.017), depletive effect of N (p < 0.001) and interaction effect (p < 0.001). Treatment l.s.d. = 0.107 μg EPS-AA nmol^−1^ ATP).

Comparison of ATP before and after extraction of EPS can provide an estimate of cell lysis (Redmile-Gordon et al., 2014). ANOVA showed no statistically significant effect of EPS extraction (Table 3; before and after p = 0.13), and only input treatment effects (p < 0.001). Microbial ATP is also a useful indicator of the size of the microbial biomass in soils recently given large amounts of labile C (Joergensen and Raubuch, 2002). EPS production efficiency (μg EPS-AA nmol^−1^ ATP; Fig. 4) provides an EPS-AA concentration per unit of soil microbial biomass as opposed to a concentration per unit soil (Section 3.3; Fig. 2b). ANOVA of treatment effects on EPS-AA concentrations attributed high statistical significance to the additive effects of C (p = 0.017), negative impact of N (p < 0.001) and an interaction between the two (p < 0.001).

## 4 Discussion

### 4.1 Comparison of extraction methods

**Fig. 5:**
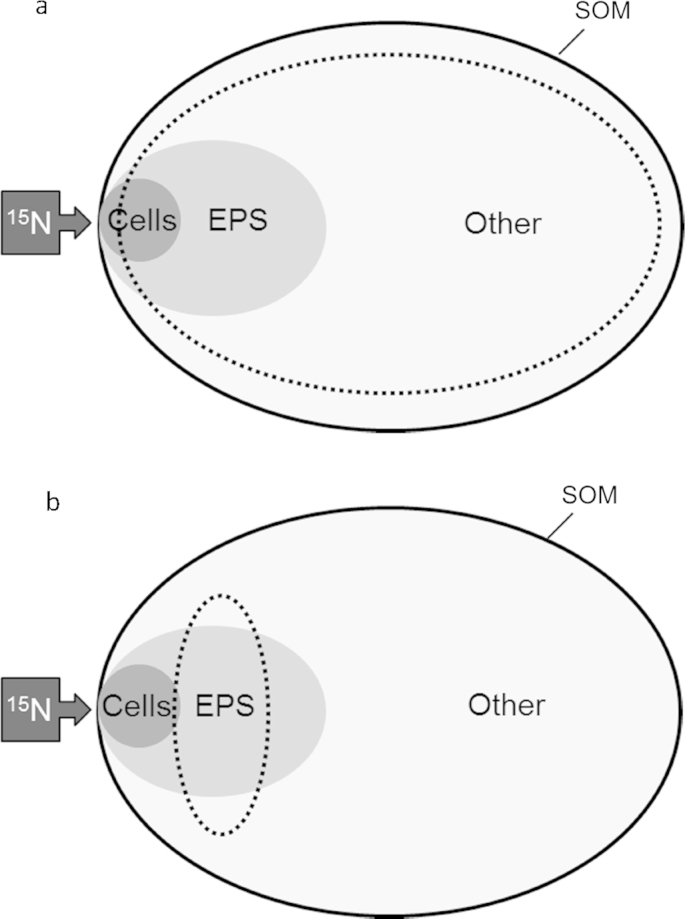
Transition of ^15^N from inorganic N to SOM. Darker shade indicates greater ^15^N enrichments contributing to the isotope signature of extracts. Dotted line indicates fraction thought to be represented by a) TSP (Jim et al., 2003), and b) EPS extracts (Redmile-Gordon et al., 2014). Not to scale.

The specificity of the EPS extraction for amino acids produced *de novo* (Fig. 1b) was confirmed by comparison to the total soil peptide extract (TSP), which contained proportionally less ^15^N-labelled AA (Fig. 1a). A ten day incubation is sufficient to allow the ^15^N label to be well-distributed throughout the majority of cellular AAs (Knowles et al., 2010). EPS exudation also tends to be greatest during the transition from exponential phases of growth into the stationary phase (Costerton et al., 1981), which probably occurred sometime after day 3. The lack of any substantial decrease in ATP following extraction (Table 3) supports the premise that EPS extraction with cation exchange resin does not cause excessive cell-lysis (Takahashi et al., 2009; Redmile-Gordon et al., 2014). Conversely, lysis from TSP extraction is extensive by design (Knowles et al., 2010). Despite this, EPS extracts still contained proportionally more ^15^N in the newly synthesised AA fraction suggesting that – unlike the TSP protocol – the majority of SOM predating application of substrate (natural abundance about 0.37% ^15^N) was not co-extracted with EPS (Fig. 5a vs. Fig. 5b). This concurs with the claim of Redmile-Gordon et al. (2014) that the cation exchange method causes minimal co-extraction of humified organic matter.

Regarding TSP, ^15^NH_4_^15^NO_3_ provided in the absence of additional C caused statistically significant ^15^N enrichment, but only within Glx. The absence of ^15^N transfer to any other AAs is strong evidence that growth of the microbial biomass was carbon-limited. Accordingly, large increases in microbial biomass ATP occurred when C was included with N (Table 3). In this case, increases in TSP ^15^N% occurred in all AAs (Fig. 1a), with Glx again becoming the most ^15^N enriched group. Assimilation of NH_4_–N occurs via two major pathways: the glutamine synthetase/glutamate synthase (GS/GOGAT) system and glutamate dehydrogenase (GDH; Payne, 1980), both resulting in the production of Glu, and thus contributing to Glx. Importantly, both GS and GDH processes are intracellular (Geisseler et al., 2009) which concurs with the current findings of highest TSP ^15^N enrichment in Glx (Fig. 1a) while Glx ^15^N enrichment was less notable in the extracts of EPS (Fig. 1b).

Quantitative analysis of TSP AAs (Section 3.3) showed no consistent effect due to treatment (Fig. 2a). In contrast, the extracted EPS-AA pool showed a consistent pattern (Fig. 2b) and statistically significant treatment effect due to the addition of C (F_1,6_ = 9.32; p = 0.022). With colorimetric analysis, the positive effect of C upon EPS-protein was even more apparent (F_1,8_ = 26.50 p < 0.001). These data support the premise that 1) the resin extraction method is more target-specific for newly biosynthesised AA's, and 2) the EPS fraction was more responsive to labile inputs than was TSP.

### 4.2 EPS dynamics

Increases in EPS-protein and EPS-AA production were observed with treatment ‘C only’ (Figs. 2b, 3 and 4). The silty loam managed historically as grassland contained a large pool of SOM (organic C and N content 2.18% and 0.204%, respectively). This SOM was relatively recalcitrant which is evident from the absence of ^15^N incorporation into TSP amino acids when ^15^N was provided without additional C (Fig. 1a). These results concur with the suggestion of Redmile-Gordon et al. (2014) that the previous absence of a statistically significant increase in EPS-protein may have occurred due to the paucity of complementary organic N in the SOM (it should also be noted that in the study of Redmile-Gordon et al. (2014) soils were of low clay content, < 8%). The present results therefore support the concept that microbiota can ‘mine’ old recalcitrant SOM for N as described by Fontaine et al. (2011).

The responses of EPS-AA concentrations (μg EPS g^−1^ soil) to added C were lower when coupled with N. Nonetheless, the provision of C and N together (treatment CN) still resulted in statistically significant ^15^N incorporation into the EPS. After calculating the biomass-specific EPS concentration (μg EPS-AA nmol ATP^−1^) a statistically significant effect of *both* C and N was observed, respectively positive, and negative (Fig. 4). This demonstrates that while C is a requirement for appreciable EPS production, the inclusion or exclusion of NH_4_NO_3_ critically affects the microbial exudation of both EPS-polysaccharide and proteinaceous EPS. The interaction observed between C and N (*p* < 0.001) demonstrates that in conditions of high C and N availability growth of the microbial biomass appears to be favoured over exudation (supported by data in Table 3).

The highest levels of EPS-polysaccharide were associated with the highest amounts of EPS-protein (Fig. 3). Polysaccharide rich EPS was also found to accumulate enzymes, preventing loss in aquatic environments (Flemming and Wingender, 2002; Romani et al., 2008). It is therefore reasonable to expect EPS also prevents losses to soil pore-water. Enzymes or peptides released freely into soil solution would subsequently become susceptible to sorptive loss onto clays and organic matter (Bastida et al., 2009; Giagnoni et al., 2013). Ducklow and Carlson (1992) also observed that maintaining exoenzymes and hydrolysis products in close proximity using an EPS system helped to keep metabolic costs low. This could be seen as a selective pressure for exudation of co-polymers to accompany the enzymes in an EPS matrix. Indeed, in freshwater systems, enzymatic activity and exudation generally do increase in tandem with phases of polysaccharide exudation (Jones and Lock, 1991; Wingender et al., 1999). Whereas these authors describe dilute hydrated environments, variably hydrated soil microenvironments will be additionally subject to peptide losses to clays and humified SOM via the ephemeral pore-water. When Miltner et al. (2009) investigated turnover of carbon from the microbial biomass they found that only circa 5% of AAs were re-metabolised from SOM whereas polysaccharides were recycled extensively.

**Fig. 6:**
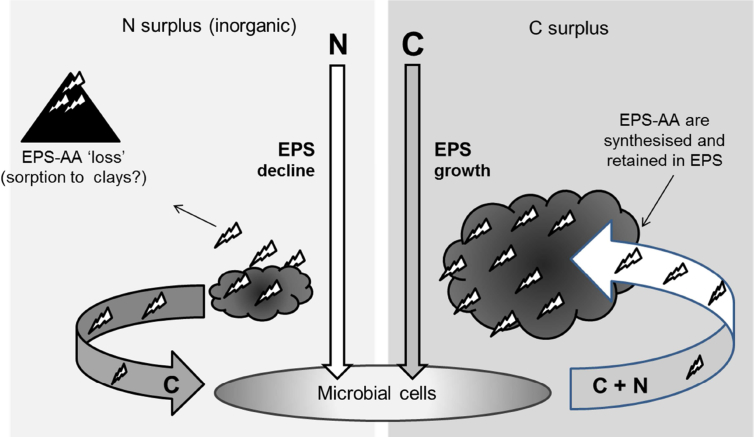
EPS dynamics in response to C:N of available substrate in soil. Microbial cells exude biopolymers rich in C (polysaccharide) and N (peptides) to confer competitive advantage in conditions of high C availability (shown right). under conditions of high inorganic N (shown left), EPS-polysaccharide is not produced/is re-metabolised and EPS-AA become susceptible to i) sorption to clays or ii) mineralisation for C acquisition to support growth of the microbial biomass.

Flemming and Wingender (2002) wrote that: “*EPS provides a template for extracellular enzymes and prevents that they are washed out... ...thus, biofilms can be considered as a natural example for sustainable use of nutrients*”. Our results, considered in light of the published works on related environments, lead us to propose a conceptual model for EPS production by the microbial biomass in soil, where C is a fundamental requirement for significant EPS production, and N acts a ‘switch’ for intra-vs extra-cellular investment of C (Fig. 6).

Fig. 6 echoes the thinking of Flemming and Wingender (2002), and fits with the observations of Miltner et al. (2009) discussed above. It reflects the issues of protein sorption to clay particles occurring when peptides are freed into soil solution (e.g. Giagnoni et al., 2013), unless in soil, as in other environments, they are protected by EPS to prevent loss to the environment (Flemming and Wingender, 2010). The results presented in the present study support this model in that i) the addition of N, either alone or in conjunction with C, resulted in lower EPS-production efficiencies, ii) EPS-polysaccharide increased most when labile C was given without N, and iii) addition of C alone caused the greatest increases in exuded EPS AAs, which is contrary to the straightforward stoichiometric expectation from the C/N ratio of inputs. To address the apparent paradox where under conditions of N deficiency, N is invested extracellularly, it should be considered that microbial cells must ‘speculate to accumulate’ i.e. produce peptides (whether structural, adhesive or enzymatic) in a way which maximises the likelihood of catalytic returns. Data obtained from investigation of the ATP costs of protein synthesis in *Escherichia coli* (Smith and Chapman, 2010), showed that the average production cost of the proteins that are secreted (including enzymes) was found to be significantly lower than the costs of *intracellular* proteins. This reflects the conservative requirement associated with the exudation of EPS-AA.

Of immediate agricultural relevance is that an increase in soil microbial EPS is thought to help extend periods of water availability in free-draining soils (Or et al., 2007b). The current data and proposed conceptual model may therefore help direct optimum N fertilisation strategies in agricultural soils affected by drought. For example, if water retention is a high priority it may be advisable to temporarily withhold N. Similarly, other managements that favour a high EPS production efficiency might be preferred. In contrast to the dynamics observed in the present study, studies in water technologies generally find a straightforward stoichiometric relationship between C/N ratio of inputs (substrate) and products (EPS) with low C/N ratios favouring proteinaceous exudation (e.g. Ye et al., 2011). However, these studies tend to use organic N as a substrate, and the dynamics in Fig. 6 relate specifically to inorganic N availability. Further studies to observe contrasts in the response of microbial EPS in soil to organic vs. inorganic sources of N would therefore be justified.

In soils, a diverse suite of temporally stable enzymes are expected to be necessary for complex hydrolysis processes. This is because to obtain N from low exergy, humified soil organic matter a cascade of catalytic processes would be required (Bosatta and Agren, 1999; Fontaine et al., 2003). Since extracellular enzymes are the primary vector for cleavage of high molecular weight organic N preceding microbial uptake (Nannipieri and Eldor, 2009; Geisseler et al., 2010), it follows that in conditions of low inorganic N availability a range of extracellular enzymes and enzyme-retentive biochemistry will be required. From a biofilm perspective an increase in EPS-AA would be an expected requirement as components of a) structural proteins which are thought to convey elasticity and resilience to the biofilm (Costerton et al., 1981), b) embedded enzymes, and c) the supporting glycoproteins or scaffoldin-like structures which can significantly improve the efficiency of hydrolysis of poorly accessible substrate (You et al., 2012).

### 4.3 SOM dynamics

The findings of the present research illuminate contrasting dynamics of confounding pools within SOM, i.e. the extracellular interface, the microbial biomass, and humified SOM. The impact of the C/N ratio of inputs on SOM destabilisation has received continued interest since early discussions by Jenkinson et al. (1985) and Kuzyakov et al. (2000). Subsequent research has assumed or ascribed a pivotal role to the microbial community in regulating the turnover of humified organic matter, and yet quantitative studies have shown there is no *direct* link between the size of the microbial biomass and mineralisation rate (Kemmitt et al., 2008). Residual extracellular enzyme activity was proposed by some to explain this phenomenon (e.g. Kuzyakov et al., 2009) but examples where attention is given to the influence of the extracellular matrix are few (e.g. Flemming and Wingender, 2002; Romani et al., 2008; Burns et al., 2013). Redmile-Gordon et al. (2014) proposed that improved understanding of organic matter dynamics could be obtained through independent measurement of the EPS.

In the present study, the N-induced increase of Glx concentration and ^15^N enrichment in TSP indicates N assimilation by the microbial biomass. This anabolic process requires energy and generally results in an efflux of CO_2_ (Geisseler et al., 2009). In the absence of light the bulk of C must be derived from cellular reserves or SOM. However, it has been proposed that while humified SOM can be a viable source of N (Fontaine et al., 2011) humified SOM alone is not a viable source of C for any increase in microbial biomass (Fontaine et al., 2007). Our results are supportive because while a measurable increase in EPS-AA's occurred with treatment ‘C only’ (suggesting N mining for AA-N) there was no substantial increase in ^15^N incorporation into other amino acids (besides Glx) with the N only treatment (Fig. 1a). Furthermore, and in respect of the mining concept, we postulate that the EPS can be an alternative source of C for microbes provided with an excess of inorganic N. This is supported by our findings that the addition of N causes a statistically significant decline in the extracellular concentration of EPS (Figs. 3 and 4). The fact that EPS was produced when C was in surplus, but decreased relative to the control when inorganic N was added suggests that, in effect, the microbial biomass may ‘store’ bioavailable C externally as EPS. We therefore term this proposed dynamic the ‘*Biorepository C hypothesis’* for future testing, which might help elucidate exceptions to the expected dynamics associated with priming effects. For example, while the addition of N does not generally result in net SOC mineralisation (Janssens et al., 2010; Chen et al., 2014) this is not without exception. Conde et al. (2005) found that the addition of NH_4_^+^ increased net mineralisation (as measured by CO_2_ emission). Interestingly, their study compared soils of variable salinity, where the greatest increases in mineralisation rates occurred in the most saline soils. Salinity induces water-deficit stress which, as described previously, invokes the survival response of increased EPS-polysaccharide production (Roberson and Firestone, 1992). Therefore, the increased CO_2_ measured by Conde et al. (2005) seems likely to have originated from N-stimulated consumption of native soil EPS as proposed in Fig. 6. Whether this had occurred or not in their study, the inclusion of EPS measures in future research will reveal if C is mineralised from the dynamic biophysical interface (EPS) or from long-dead humified SOM. Accordingly, in addition to the rationalisation presented by Blagodatskaya and Kuzyakov (2008), to prevent confounding these pools we suggest that CO_2_ from the mineralisation of EPS should not be considered a component of the ‘true priming effect’.

The speculate-to-accumulate principle discussed in Section 4.2 is also supported by contemporary investigations into SOM dynamics. Chen et al. (2014) found that with sole C treatments, a positive priming effect (mineralisation of SOM coupled with enzyme production) was accompanied by a decrease in cell-specific growth rates. From the ATP concentrations given in Table 3 it is seen that also here the addition of C alone did not result in the accumulation of microbial biomass (unlike treatment CN). While Chen et al. (2014) explained their slow growth rate as evidence of a greater contribution from *K-strategists* decomposing the native SOM, our new perspective suggests that decreased cellular growth could be explained by the increased exudation of extracellular polysaccharides and peptides (Fig. 3). This increased EPS-production efficiency is postulated here to be an investment strategy to maximise enzyme efficiency by retention of enzymes in a polysaccharide-rich matrix, maintaining proximity (and thus efficiency) for the producing cells (Fig. 6). While this hypothesis requires further testing over time, the amino acid profiles (Figs. 2b and 4) currently support it.

Within the TSP profiles (Fig. 2a) – the seven most abundant AAs (ala, gly, pro, thr, asx, ser, gsx) appeared to become depleted with imbalanced substrate provision (treatment C or N alone). The importance of these AA residues in recalcitrant organic matter is supported by virtue of their abundance in the extract. The same AA residues were also found to be dominant in previous studies on grassland and arable soils with contrasting histories (e.g. Friedel and Scheller, 2002; Jämtgård et al., 2010). This raises the question: why are these residues more abundant? The intrinsic chemical recalcitrance of individual AAs themselves does not predict abundance in SOM because the half-lives of AAs added to soils do not correlate with the abundances of the old hydrolysable pools observed here (see Farrell et al., 2014). These discrepancies could be due to increased recalcitrance through biogenic association (e.g. glycosylation; Sola et al., 2006), stabilisation on soil reactive surfaces (Nannipieri et al., 2012), complexation with phenolic compounds (Mazzei et al., 2013) or simply due to being physically inaccessible (Darrouzet-Nardi and Weintraub, 2014). However, returning focus to the data presented in Fig. 2a, the decrease in response to C provision (alone) again adds support to the concept of ‘N mining’ discussed by Fontaine et al. (2011). This depletion in TSP-AAs was accompanied by an increase in measurable EPS-AAs (Fig. 2b) which incorporated non-labelled N (Fig. 1b).

The decrease in the most abundant AAs in TSP in response to N provision (alone) may be seen to suggest the SOM AAs were also mined for C, however, there was no corresponding increase in either microbial biomass or EPS, which again supports the assertion of Fontaine et al. (2007) that humified SOM is not a viable source of C for significant microbial growth. In summary, the abundant groups' trend for decline with provision of unbalanced substrate (C without N, or N without C) contrasted with that of the less abundant group which instead showed increases in response to C as seen with the EPS-AA pool (Fig. 2b). It is not known what underlies this division in TSP-AA profile response to labile inputs because the pools within TSP cannot be distinguished. Nonetheless, the post-hoc analysis showed that the large and small subgroups were indeed responding differently.

### 4.4 Amino acids showing high relative abundance in EPS

Ala, Gly, Pro, Phe and Tyr all showed marked increases in relative abundance of EPS compared to TSP. Friedel and Scheller (2002) also found increased relative abundance of Phe and Tyr outside microbial cells. Besides Gly, which can fit into both hydrophilic and hydrophobic domains, all these residues have hydrophobic side-chains. Also, besides Gly, all have high C/N ratios which supports previous work stating that the expressed protein C/N ratio adapts to suit the environment (Bragg and Wagner, 2007). We found Thr, Asx and Ser concentrations were notably lower in EPS than in TSP. Friedel and Scheller (2002) attributed high Asx and Ser to contributions from fungal cell walls (Wagner and Mutatkar, 1968). The large increases in relative abundances of Pro and Gly in EPS suggest structural importance, both fulfilling formative roles in collagen and implicated in the adhesion between *Trichodesmium erythraeum* and heterotrophic bacteria in the marine environment (Price et al., 2014). Proline and glycine rich domains also impart elasticity to elastin (Le et al., 2013) and the glycoprotein fibrillin (Mariko et al., 2010). Costerton et al. (1981) observed that extracellular fibrils were a common feature of many EPS, forming a ‘helical duplex’ around the producing organisms. Biofilms increase in tensile strength as matric potential decreases (Or et al., 2007b). Therefore, as matric potential fluctuates over wet/dry cycles in soil, cells retained in EPS will be a) less susceptible to translocation and dispersal, and b) able to take advantage of the greater mass of potential solute exchange (afforded by the increased differential between pore-water flux and the anchored cell-clusters). It seems reasonable therefore that the present experimental design, which incorporated wet/dry cycles, may have contributed to the abundance of these structural amino acids with hydrophobic side-chains.

From an enzyme perspective, the role of proline is especially intriguing as it conveys unique properties of mobility and conformational flexibility to intrinsically disordered proteins (Lee et al., 2014). Enzymes featuring intrinsic disorder possess increased mobility and challenge the traditional ‘lock-and-key’ paradigm enabling more promiscuous behaviour in target interactions (Uversky and Dunker, 2010). Intrinsic disorder has also been observed in scaffoldins (Hammel et al., 2005) and proline is also abundant in scaffoldin components of EPS (Slutzki et al., 2013). Scaffoldins were demonstrated to improve the hydrolysis efficiency of poorly accessible substrate (You et al., 2012). Scaffoldin modules also serve to bind the producing cells to the target substrates (Fontes and Gilbert, 2010). These properties are almost certainly important in the degradation of SOM, both due to the chemical diversity of SOM, and due to the physical occlusion of substrates in soil (e.g. Six et al., 2004; Schmidt et al., 2011).

### 4.5 Conclusions

Cation exchange resin preferentially extracts peptides in the EPS produced *de novo* over the peptides in the older and more ambiguous humified SOM pool. In this recently converted arable soil containing a large residual organic N pool, colorimetrically determined EPS-protein and EPS-polysaccharide increased through provision of labile C without the addition of any exogenous N. GC-FID analysis supported these observations by quantification of the extracellular amino acid pool. This adds support to Fontaine et al.'s work (2011) proposing that microbes utilise N from humified soil organic matter. Studies linking EPS dynamics with priming effects are now required to determine potential roles of EPS in SOM turnover.

When inorganic N was provided, the EPS production efficiency (μg EPS nmol^−1^ ATP) declined substantially indicating that surplus inorganic N downregulates EPS production. When C was provided the EPS production efficiency increased. More studies into the underlying triggers for expression of the EPS phenotype are clearly needed. Understanding these triggers will enable harmonisation of soil management with native EPS dynamics and thus help deliver management objectives more sustainably.

## Figures and Tables

**Table 1:** Treatment quantities added to each microcosm of moist soil.

Treatment name	Glycerol C (mg)	NH_4_NO_3_ (mg)	N (mg)	Labile C/N	H_2_O (mL)
0	–	0	0	–	4.93
C	99	0	0	>100	4.93
N	–	14.14	4.95	<1	4.93
CN	99	14.14	4.95	20	4.93

**Table 2:** Mean extract amino acid (AA) concentration. Individual data transformed log_10_ for ANOVA (means of logs presented for statistical comparison).

Treatment	TSP-AA (μg g^−1^ soil)	TSP-AA (μg g^−1^ soil log_10_)	EPS-AA (μg g^−1^ soil)	EPS-AA (μg g^−1^ soil log_10_)
0	382	2.35^x^	5.29	0.533^ab^
C	359	2.35^x^	6.75	0.620^a^
N	347	2.32^x^	4.76	0.492^bc^
CN	433	2.41^x^	6.12	0.598^a^

ANOVA statistically significant effect for C (EPS only) p = 0.02; l.s.d log_10_ = 0.077; means with same letter are not statistically different.

**Table 3:** Microbial EPS concentration (based upon biomass-ATP and colorimetric analyses of EPS extracts).

Treatment	ATP before EPS extraction* (nmol g^−1^ soil)	ATP after EPS extraction* (nmol g^−1^ soil)	EPS-polysaccharide (μg nmol^−1^ ATP)	EPS-protein (μg nmol^−1^ ATP)
0	5.42 ± 0.13^b^	4.61 ± 0.59^b^	86.55^y^	32.56^f^
C	4.09 ± 0.74^b^	4.72 ± 0.16^b^	143.92^**x**^	49.95^**e**^
N	5.27 ± 0.51^b^	4.37 ± 0.46^b^	91.54^y^	33.42^f^
CN	11.84 ± 3.18^**a**^	9.69 ± 0.59^**a**^	48.12^z^	19.79^g^

^ab xyz efg^ means with same letter are not statistically different. l.s.d's _(α=0.05)_: ATP treatment effect = 1.60 nmol g^−1^ soil; EPS-polysaccharide = 21.6 μg nmol^−1^ ATP; EPS-protein = 8.7 μg nmol^−1^ ATP.

* No statistically significant effect of comparison ATP before vs. after EPS extraction (P = 0.13).
